# Association of Open Approach vs Laparoscopic Approach With Risk of Surgical Site Infection After Colon Surgery

**DOI:** 10.1001/jamanetworkopen.2019.13570

**Published:** 2019-10-18

**Authors:** Daniel A. Caroff, Christina Chan, Ken Kleinman, Michael S. Calderwood, Robert Wolf, Elizabeth C. Wick, Richard Platt, Susan Huang

**Affiliations:** 1 Department of Population Medicine, Harvard Medical School and Harvard Pilgrim Health Care, Boston, Massachusetts; 2 now with Department of Infectious Diseases, Lahey Hospital and Medical Center, Burlington, Massachusetts; 3 Department of Biostatistics and Epidemiology, University of Massachusetts Amherst School of Public Health and Health Sciences, Amherst; 4 Section of Infectious Disease & International Health, Dartmouth-Hitchcock Medical Center, Lebanon, New Hampshire; 5 now with Boston University School of Medicine, Boston, Massachusetts; 6 Division of General Surgery, University of California, San Francisco; 7 Division of Infectious Diseases and the Health Policy Research Institute, University of California Irvine School of Medicine, Irvine

## Abstract

**Importance:**

Colon surgery is associated with a high rate of surgical site infection (SSI), and there is an urgent need for strategies to reduce infection rates.

**Objective:**

To assess whether laparoscopic colon surgery is associated with a lower surgical site infection rate than open-approach laparoscopy, especially in patients with medically complex conditions.

**Design, Setting, and Participants:**

This cohort study used previously validated diagnosis and procedure codes from Medicare beneficiaries who underwent colon surgery from January 1, 2009, to November 30, 2013. Analyses were performed from August 1 to December 31, 2018.

**Main Outcomes and Measures:**

Outcome measures were SSI events, medical comorbidities, and laparoscopic or open approach procedures.

**Results:**

A total of 229 726 patients (mean [SD] age, 74.3 [9.4] years; 128 499 [55.9%] female) underwent colon procedures. There were 105 144 laparoscopic procedures and 124 582 open procedures. The overall mean SSI rate was 6.2%, varying by surgical procedure from 5.8% to 7.6%. Among the full study population, adjusted model results showed a significant association of laparoscopy with lower odds of SSI (odds ratio, 0.43; 95% CI, 0.41-0.46; *P* < .001). When stratified by surgical approach, the mean SSI rates were 4.1% (procedure-specific range, 3.9%-5.1%) for the laparoscopic approach and 7.9% (procedure-specific range, 7.4%-10.2%) for the open approach. When stratified by Elixhauser score groups, the mean SSI rates were 6.2% (procedure-specific range, 3.2%-8.7%) for group 1 (0-1 comorbidity), 5.5% (procedure-specific range, 3.6%-11.1%) for group 2 (2 comorbidities), and 6.6% (procedure-specific range, 4.6%-10.6%) for group 3 (3-13 comorbidities). An interaction was also observed between laparoscopic approach and Elixhauser groups, with increased odds of SSI among patients who had 3 to 13 comorbidities present at the time of the procedure (odds ratio, 1.21; 95% CI, 1.11-1.32) compared with patient groups with fewer comorbidities. The population attributable fraction of SSIs for use of the open approach was 34.2%. A total of 2317 of 3882 hospitals (59.7%) performed few (0%-10%) or most (>50%) procedures laparoscopically.

**Conclusions and Relevance:**

Policy changes that promote surgical education and resources for laparoscopy, especially at low-adoption hospitals, may be associated with reduced colon SSI rates. Support of the development of innovative educational policies may help achieve improvement in patient outcomes and decreased health care use in colon surgery.

## Key Points

**Question:**

Is laparoscopic colon surgery associated with a lower surgical site infection rate than an open approach, even in patients with high medical complexity?

**Findings:**

In this cohort study of 229 726 patients undergoing colon operations, compared with an open approach, laparoscopic colon surgery was associated with a lower surgical site infection rate regardless of medical comorbidities. Patients with multiple comorbidities underwent open colon surgery more often than laparoscopy.

**Meaning:**

Increasing the use of laparoscopy for colon surgery may be associated with reduced risk of surgical site infection.

## Teaser

This cohort study compares the surgical site infection rate associated with laparoscopic colon surgery vs open-approach laparoscopy.

## Introduction

Surgical site infections (SSIs) are associated with substantial morbidity, often requiring additional operation and unanticipated hospitalization, with estimated attributable costs of more than $3 billion annually.^1,2^ Preventing infection after colon surgery is a national priority for the more than 300 000 procedures performed annually in the United States,^3^ with their associated SSI rates being as high as 14% to 25%.^4,5^ Despite advances in understanding processes for colon SSI prevention and adoption of bundles of processes, the SSI rate after colon surgery continues to be high and varies considerably among hospitals, suggesting that further improvement is possible.^6^

Laparoscopic colon surgery, first described in the 1990s, is a safe alternative to traditional open surgery for many patients.^7^ Laparoscopy, in addition to accelerating recovery after colon surgery by decreasing pain and duration of hospital stay, also has been shown to be associated with a lower risk of SSI.^4,5^ Despite these benefits, many patients undergo open colon surgery for various reasons, some surgeon specific and some patient specific. A patient’s general health may influence the decision regarding surgical approach; patients undergoing a laparoscopic procedure tend to be younger and fitter and have significantly fewer comorbidities.^5^ In addition, the complexity of the underlying surgical condition and prior surgical procedures may make laparoscopy more challenging. However, many surgeons reliably perform laparoscopic surgery for patients with both medically and surgically complex conditions.^8^ The reasons behind and strategies to address the continued variation in uptake of laparoscopic colorectal surgery continue to be debated. Professional societies have developed programs to assist surgeons in practice with transitioning their approach to colon surgery from open to laparoscopic,^9^ and general and colorectal residency review committees have prioritized competency in laparoscopy for trainees.^10^

The association of addressing the variation in adoption of laparoscopic colon surgery with colon SSI rates in the United States remains unclear. Therefore, we sought to study the distribution of SSI risk among patients undergoing analogous open and laparoscopic colon surgery, stratified by comorbidity status.

## Methods

### Study Design and Cohort Selection

In this cohort study, we evaluated patient-level SSI rates after colon surgery among fee-for-service Medicare beneficiaries older than 18 years between January 1, 2009, and November 30, 2013, using Medicare Provider Analysis and Review, a deidentified database of Medicare beneficiaries who used hospital inpatient services.^11,12^ We identified 508 140 colon operations using the 37 *International Classification of Diseases, Ninth Revision (ICD-9)* procedure codes used by the Centers for Disease Control and Prevention and Centers for Medicare & Medicaid Services for national SSI surveillance and reporting.^13^ We considered 7 types of paired colon operations (ie, those with claims codes for both open and laparoscopic approaches) (eAppendix in the Supplement). We excluded 277 284 procedures performed with a concomitant intra-abdominal or other colon surgery and 3 procedures performed in patients younger than 18 years. For each instance of colon surgery, we identified patient descriptors, including age, sex, race/ethnicity, and claims-based Elixhauser comorbidities^14^ present at the time of surgery. We followed the Strengthening the Reporting of Observational Studies in Epidemiology (STROBE) reporting guideline for cohort studies. This study was approved by the Harvard Pilgrim Health Care’s Institutional Review Board, which provided a waiver of consent. All data were deidentified.

### Definition of SSI

The SSI determinations were made by using previously validated administrative claims codes that indicated postsurgical infection.^12^ These codes accurately rank hospitals by their SSI rates.^15^ We assessed claims within 30 days of the surgical procedure for administrative claims codes suggestive of a deep or organ/space SSI based on *ICD-9* procedure codes 54.0, 54.11, 54.19, 86.04, 86.22, and 86.28 and *ICD-9* diagnostic codes 567.21, 567.22, 567.29, 567.38, 569.5, 569.61, 569.81, 682.2, 879.9, 998.31, 998.59, and 998.6. To address preexisting infections, we excluded procedures in which an SSI claims code was designated as present on admission during the index surgical hospitalization. For patients who underwent another major surgery in the 30-day postoperative surveillance period, we censored our surveillance at the time of the subsequent surgery.

### Definition of Elixhauser Comorbidities

We categorized patients by the number of Elixhauser comorbidities (group 1: 0-1 comorbidities, group 2: 2 comorbidities, and group 3: 3-13 comorbidities) and grouped them by the surgical procedure that they underwent.

### Statistical Analysis

We compared SSI rates between patients treated by the laparoscopic vs open approach for each surgical procedure, stratified by Elixhauser category. We then ran a logistic regression model to assess the association of surgical approach with SSI, adjusting for procedure type, age, sex, race/ethnicity, and number of Elixhauser comorbidities. These covariates were preselected for inclusion in the model based on clinical judgment. We also assessed an interaction term between comorbidity status and approach. The logistic models were run using generalized estimating equations to account for clustering across hospitals. We repeated this logistic model individually for each type of surgical procedure.

We calculated the population attributable fraction defined as the proportion of SSIs that can be attributed to having had an open procedure for the entire population, both for the full population and for each procedure. We then graphed the proportion of each hospital’s procedures that were performed using a laparoscopic approach. All analyses were performed from August 1 to December 31, 2018, using SAS statistical software, version 9.4 (SAS Institute Inc). Statistical significance was set at 2-sided *P* < .05, using Wald 95% CIs.

## Results

Among 7 paired colon operations, 230 853 procedures were eligible for inclusion. Two surgical procedures (multiple segmental resection of large intestine and total intra-abdominal colectomy) were only performed laparoscopically and were excluded from analysis. Among the remaining 5 paired surgical procedures, 229 726 procedures were identified among 3882 hospitals, including right hemicolectomy, left hemicolectomy, sigmoidectomy, other partial excision of the large intestine or cecectomy, and transverse colectomy, all performed by laparoscopy and an open approach (Table 1). Therefore, a total of 229 726 patients (mean [SD] age, 74.3 [9.4] years; 128 499 [55.9%] female) underwent colon procedures. There were 105 144 laparoscopic procedures and 124 582 open procedures. Procedures for patients in Elixhauser groups 1 and 2 were performed more often by laparoscopic approach, whereas procedures for patients in Elixhauser group 3 were performed more often by open approach. The overall mean SSI rate was 6.2%, varying by surgical procedure from 5.8% to 7.6%. When stratified by surgical approach, the mean SSI rates were 4.1% (procedure-specific range, 3.9%-5.1%) for the laparoscopic approach and 7.9% (procedure-specific range, 7.4%-10.2%) for the open approach. When stratified by Elixhauser score groups, the mean SSI rates were 6.2% (procedure-specific range, 3.2%-8.7%) for group 1, 5.5% (procedure-specific range, 3.6%-11.1%) for group 2, and 6.6% (procedure-specific range, 4.6%-10.6%) for group 3.

**Table 1.** Characteristics of Colectomy Procedures

Characteristic	Right Hemicolectomy		Sigmoidectomy		Left Hemicolectomy		Other Partial Excision of Large Intestine and Cecectomy		Resection of Transverse Colon	
	Laparoscopic	Open	Laparoscopic	Open	Laparoscopic	Open	Laparoscopic	Open	Laparoscopic	Open
No. of patients (n = 229 726)	55 871	65 194	30 541	35 218	8138	13 434	6772	4472	3822	6264
No. of operative hospitals	2809	3532	2514	3341	1847	2798	1808	1894	1373	2180
No. of procedures per performing hospital, mean (SD)	19.89 (27.02)	18.46 (20.90)	12.15 (15.11)	10.54 (11.04)	4.41 (5.25)	4.80 (4.89)	3.75 (4.28)	2.36 (1.87)	2.78 (2.60)	2.87 (2.52)
Patient age, mean (SD), y	74.86 (8.54)	75.90 (9.59)	71.55 (8.80)	73.18 (9.79)	73.40 (8.75)	74.28 (9.46)	72.93 (9.44)	73.84 (11.21)	74.75 (8.49)	75.88 (9.61)
Female, No.(%)	30 243 (54.1)	37 222 (57.1)	17 822 (58.4)	20 029 (56.9)	4199 (51.6)	7045 (52.4)	3696 (54.6)	2654 (59.4)	1934 (50.6)	3655 (58.4)
Total Elixhauser score, mean (SD)	2.14 (1.60)	2.45 (1.74)	1.82 (1.46)	2.11 (1.59)	2.08 (1.56)	2.28 (1.64)	1.92 (1.57)	2.28 (1.78)	2.13 (1.68)	2.41 (1.70)
Overall No. of infections, mean (SD)	2156 (3.9)	4857 (7.4)	1265 (4.1)	2884 (8.2)	419 (5.1)	1225 (9.1)	262 (3.9)	455 (10.2)	157 (4.1)	467 (7.5)
Elixhauser group 1 (n = 87 214)^a^										
No. of procedures, mean (SD)	21 616 (38.7)	21 010 (32.2)	14 378 (47.1)	13 921 (39.5)	3221 (39.6)	4691 (34.9)	3082 (45.5)	1717 (38.4)	1541 (40.3)	2037 (32.5)
No. of infections, mean (SD)	843 (3.9)	1705 (8.1)	569 (4)	1158 (8.3)	169 (5.2)	442 (9.4)	111 (3.6)	191 (11.1)	64 (4.2)	164 (8.1)
Elixhauser group 2 (n = 57 689)^a^										
No. of procedures, mean (SD)	14 477 (25.9)	15 710 (24.1)	7870 (25.8)	8865 (25.2)	2174 (26.7)	3410 (25.4)	1682 (24.8)	1006 (22.5)	967 (25.3)	1528 (24.4)
No. of infections, mean (SD)	487 (3.4)	1036 (6.6)	293 (3.7)	675 (7.6)	92 (4.2)	289 (8.5)	60 (3.6)	85 (8.4)	26 (2.7)	108 (7.1)
Elixhauser group 3 (n = 84 823)^a^										
No. of procedures, mean (SD)	19 778 (35.4)	28 474 (43.7)	8293 (27.2)	12 432 (35.3)	2743 (33.7)	5333 (39.7)	2008 (29.7)	1749 (39.1)	1314 (34.4)	2699 (43.1)
No. of infections, mean (SD)	826 (4.2)	2116 (7.4)	403 (4.9)	1051 (8.5)	158 (5.8)	494 (9.3)	91 (4.5)	179 (10.2)	67 (5.1)	195 (7.2)

^a^ Group 1 indicates 0 to 1 comorbidities; group 2, 2 comorbidities; and group 3, 3 to 13 comorbidities.

Among the full study population, adjusted model results showed a significant association of laparoscopy with lower odds of SSI (odds ratio, 0.43; 95% CI, 0.41-0.46; *P* < .001). An interaction was also observed between laparoscopic approach and Elixhauser groups, with increased odds of SSI among patients who had 3 to 13 comorbidities present at the time of the procedure (odds ratio, 1.21; 95% CI, 1.11-1.32) compared with patient groups with fewer comorbidities (Table 2). Models for individual surgical procedures showed that this interaction was associated with patients undergoing right hemicolectomy and sigmoidectomy; the interaction was not significant for the other 3 procedures (Table 3). All individual models showed a protective association of laparoscopy with risk of SSI.

**Table 2.** Overall Multiple Logistic Regression Model on Surgical Site Infection Using Generalized Estimating Equations

Variable	Odds Ratio (95% CI)
Age (continuous)	0.99 (0.99-0.99)
Female	0.78 (0.76-0.81)
Race/ethnicity	
White	1 [Reference]
Black	1.03 (0.97-1.09)
Other	1.05 (0.91-1.22)
Asian	0.97 (0.80-1.18)
Hispanic	1.31 (1.14-1.51)
North American Native	1.65 (1.35-2.02)
Unknown	1.02 (0.78-1.33)
No. of claims-based Elixhauser comorbidities	
0-1	1 [Reference]
2	0.86 (0.81-0.91)
3-13	0.97 (0.93-1.02)
Laparoscopic approach	0.43 (0.41-0.46)
Laparoscopy × Elixhauser group	
0-1	1 [Reference]
2	1.05 (0.95-1.16)
3-13	1.21 (1.11-1.32)

**Table 3.** Multiple Logistic Regression Models by Surgical Procedure Using Generalized Estimating Equations

Variable	Odds Ratio (95% CI)				
	Right Hemicolectomy	Resection of Transverse Colon	Left Hemicolectomy	Sigmoidectomy	Other Partial Excision of Large Intestine and Laparoscopic Cecectomy
Age, continuous	0.99 (0.98-0.99)	1.00 (0.99-1.01)	0.99 (0.99-1.00)	0.99 (0.99-1.00)	0.98 (0.98-0.99)
Female	0.72 (0.69-0.76)	0.71 (0.60-0.83)	0.83 (0.75-0.92)	0.90 (0.84-0.95)	0.78 (0.67-0.91)
Race/ethnicity					
White	1 [Reference]	1 [Reference]	1 [Reference]	1 [Reference]	1 [Reference]
Black	1.00 (0.92-1.09)	1.39 (1.09-1.77)	1.04 (0.89-1.21)	1.07 (0.95-1.21)	1.03 (0.80-1.33)
Other	1.16 (0.95-1.42)	0.46 (0.16-1.26)	1.03 (0.69-1.53)	1.11 (0.86-1.43)	0.80 (0.38-1.68)
Asian	0.91 (0.69-1.21)	0.94 (0.46-1.94)	1.21 (0.84-1.75)	1.07 (0.78-1.45)	0.72 (0.30-1.75)
Hispanic	1.53 (1.29-1.82)	0.89 (0.40-1.99)	1.15 (0.78-1.71)	1.21 (0.95-1.54)	0.69 (0.31-1.56)
North American Native	1.60 (1.18-2.15)	4.11 (2.11-8.02)	1.87 (0.94-3.71)	1.39 (0.94-2.06)	1.41 (0.50-3.99)
Unknown	0.92 (0.60-1.39)	0.97 (0.24-3.96)	0.50 (0.16-1.60)	1.56 (1.06-2.31)	0.24 (0.03-1.86)
No. of claims-based Elixhauser comorbidities					
0-1	1 [Reference]	1 [Reference]	1 [Reference]	1 [Reference]	1 [Reference]
2	0.81 (0.75-0.88)	0.89 (0.69-1.14)	0.90 (0.77-1.05)	0.92 (0.84-1.01)	0.76 (0.58-0.99)
3-13	0.93 (0.87-1.00)	0.90 (0.72-1.12)	1.00 (0.87-1.14)	1.04 (0.95-1.13)	0.96 (0.77-1.20)
Laparoscopic approach	0.44 (0.40-0.48)	0.49 (0.36-0.65)	0.53 (0.44-0.63)	0.45 (0.40-0.50)	0.29 (0.23-0.38)
Laparoscopy × Elixhauser group					
0-1	1 [Reference]	1 [Reference]	1 [Reference]	1 [Reference]	1 [Reference]
2	1.08 (0.93-1.24)	0.72 (0.42-1.23)	0.90 (0.66-1.23)	1.04 (0.87-1.23)	1.31 (0.87-1.99)
3-13	1.19 (1.06-1.34)	1.38 (0.91-2.09)	1.12 (0.86-1.46)	1.22 (1.04-1.42)	1.39 (0.98-1.97)

The SSI rates were consistently lower for each surgical procedure when performed laparoscopically (aggregate SSI rate, 3.9%) compared with the open approach (aggregate SSI rate, 7.9%); this association was consistent in each Elixhauser score category (Table 4). The population attributable fraction of SSIs associated with use of the open approach was 34.2% (range by procedure, 32.4%-39.3%) (Table 4).

**Table 4.** Population Attributable Fractions and Raw SSI Rates, Stratified by Surgical Approach (Laparoscopic or Open)

Procedure	No. (% Unadjusted SSI Rate)			PAF for Open Procedures, %
	Overall	Laparoscopic	Open	
Total, all 5 procedures combined	229 726 (6.2)	10 5144 (4.1)	124 585 (7.9)	34.2
Right hemicolectomy	121 065 (5.8)	55 871 (3.9)	65 194 (7.4)	33.4
Sigmoidectomy	65 759 (6.3)	30 541 (4.1)	35 218 (8.2)	34.4
Left hemicolectomy	21 572 (7.6)	8138 (5.1)	13 434 (9.1)	32.4
Other partial excision of large intestine	11 244 (6.4)	6772 (3.9)	4472 (10.2)	39.3
Resection of transverse colon	10 086 (6.2)	3822 (4.1)	6264 (7.5)	33.6

Abbreviations: PAF, population attributable fraction; SSI, surgical site infection.

The percentage of laparoscopic cases by hospital followed a bimodal distribution, with 2317 of 3882 hospitals (59.7%) performing few (0%-10%) or most (>50%) operations laparoscopically (Figure). Hospitals most commonly performed 0% to 10% of their colon procedures laparoscopically. The exception to this trend was other partial excision of large intestine or cecectomy, which in 1504 of 2465 hospitals (61.0%) was performed by laparoscopy more often than by the open approach.

**Figure.**
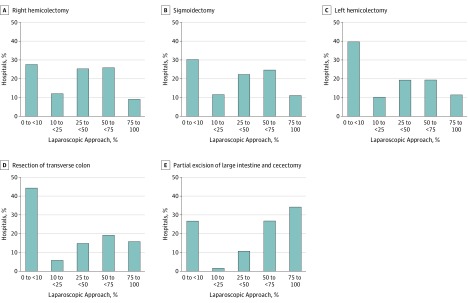
Percentage of Hospitals Using the Laparoscopic Approach for Colectomy

## Discussion

Despite considerable efforts with SSI prevention bundles, many hospitals continue to have higher than expected SSI rates associated with colon surgery.^16^ For most procedures, laparoscopy is substantiated by evidence and has been embraced by the surgical community, but the uptake in practice continues to be variable.^6^ For colon surgery, there are benefits to the laparoscopic approach, including an association with reduced risk of SSI and health care utilization as well as improvement in patient experience and patient-reported outcomes. In a large population of Medicare patients undergoing colon surgery, most patients with conditions of low medical complexity underwent operation with a laparoscopic approach, but patients with conditions of greater medical complexity more often had operations with an open approach. However, we found that use of laparoscopy for colon surgery was associated with lower SSI rates, regardless of how many chronic health conditions were present or what type of colectomy was performed. We found evidence of an interaction between laparoscopy and Elixhauser score among right hemicolectomies and sigmoidectomies, suggesting that multiple comorbidities may be associated with an increased risk of SSI among certain patients undergoing laparoscopic procedures. Despite this finding, the laparoscopic approach seems to be associated with a lower rate of SSI compared with the open approach even in patients with more medically complex conditions, and there is continued opportunity to expand the adoption of laparoscopic colon surgery and potentially reduce the SSI rate seen across all colon procedures.

Of interest, unlike a prior study^6^ evaluating the adoption of laparoscopic colectomy in the United States, we identified a bimodal pattern with the laparoscopic approach. For all types of colectomies except ileocecectomies, a cluster of hospitals completed less than 10% of their colectomies laparoscopically, and another group used the approach in 50% to 75% of their colectomies. This finding suggests that there might be a point at which laparoscopy becomes the standard approach at a hospital. This point may be related to having skilled assistants, appropriate equipment, or the presence of partners who can support skill development. When a hospital's culture or policies shift toward performing most colectomies laparoscopically, the referral patterns may evolve such that patients who need these procedures are preferentially referred to a surgeon who offers laparoscopy. This important finding needs additional study because it has implications for policy interventions to accelerate laparoscopy at the low-adoption hospitals.

In our multivariable model, in addition to the laparoscopic approach, age appeared to be associated with lower risk of SSI. This finding may be a reflection of the diseases that require colon surgery in younger vs older patients. Most colectomies in older patients are performed for cancer, and this subset of patients have a lower SSI rate compared with those who undergo colectomy for diverticulitis and inflammatory bowel disease.^5^ In addition, we found an increased SSI risk among Hispanic and Native American patients vs other races/ethnicities.

Our data indicate that one-third of all colon SSIs in this study’s population may have been associated with use of open surgery. We do not assert that each of these infections would have been avoided simply by changing surgical approach because many other patient-level factors may contribute to SSI risk. However, we found that rates of laparoscopy continued to vary significantly across hospitals. These differences were not fully explained by case mix and could have reflected differences in surgeon training, preference in surgical approach, and/or available resources at the hospital level. Thus, increasing training and preference for laparoscopic approaches may modify SSI risk to a greater capacity than previously thought, especially in patients with multiple comorbidities who currently tend to undergo open procedures in many hospitals.

### Limitations

This study has limitations. Administrative data have limitations with regard to the indication for surgery and the complexity of the surgical procedure. For example, emergency procedures may be less appropriate for laparoscopy, and we did not have data on whether procedures were urgent. Such patients may require surgery in the middle of the night, and resources for laparoscopy may be limited. Patients with bowel obstructions, free perforation, or complex inflammation are challenging to treat and require advanced laparoscopic skills. We were not able to identify prior operations and the degree of adhesions that a patient may have, a factor well established to influence surgical approach planning. The sensitivity of claims codes for identification of SSI surveillance is imperfect, yet these codes have been nationally validated and represent an innovative approach that allows for large populations to be evaluated, which would not be possible with traditional surveillance approaches. In patients with colorectal disease specifically, claims codes have a sensitivity of 84% vs 21% using traditional surveillance methods.^12^ We did not have data to adjust for hospital-level rates of laparoscopy for factors that may contribute to the differences seen in the Figure. Also, we recognize that surgeons who are inexperienced with laparoscopy cannot adopt laparoscopic techniques without significant additional training. It is incumbent on training programs to ensure proficiency in laparoscopy, for colorectal surgeons trained in laparoscopy to maximize its use in practice, and for the health care delivery system to facilitate its adoption. Professional societies and the residency review committee for surgery have worked to fill the training gap through mentoring and other innovative approaches as well as residency training requirements. Additional efforts are underway to understand the role that peer coaching may have in advancing skills in laparoscopic colectomy and promoting greater adoption.^17^

## Conclusions

The findings suggest that laparoscopic colon surgery is associated with a lower SSI rate than open colon surgery in both relatively healthy patients and those with multiple comorbidities. Patients with greater medical complexity were more likely to undergo open colon surgery, which is associated with a greater risk of SSIs than with use of laparoscopy. Although adoption of laparoscopy for colon surgery has progressed, there continues to be opportunity to increase its use. To achieve improvement in patient outcomes and decreased health care utilization in colon surgery, innovative educational policies appear to be needed.

## Notes

Supplement. **eAppendix.** Surgical Procedures Considered and Corresponding *International Classification of Diseases, Ninth Revision* (*ICD-9*) Procedure Codes
