# Supplementary material for: Association of Open Approach vs Laparoscopic Approach With Risk of Surgical Site Infection After Colon Surgery
Source: JAMA Netw Open. 2019 Oct 18;2(10):e1913570. doi: 10.1001/jamanetworkopen.2019.13570 (PMC6813583; doi:10.1001/jamanetworkopen.2019.13570)
Supplement: Supplement. — eAppendix. Surgical Procedures Considered and Corresponding International Classification of Diseases, Ninth Revision (ICD-9) Procedure Codes [file jamanetwopen-2-e1913570-s001.pdf]

Supplementary Online Content

Caroff DA, Chan C, Kleinman K, et al. Association of open approach vs laparoscopic approach with risk of surgical site infection after colon surgery. *JAMA Netw Open*. 2019;2(10):e1913570. doi:10.1001/jamanetworkopen.2019.13570

eAppendix. Surgical Procedures Considered and Corresponding *International Classification of Diseases, Ninth Revision* (*ICD-9*) Procedure Codes

This supplementary material has been provided by the authors to give readers additional information about their work.

© 2019 Caroff DA et al. *JAMA Network Open.*

eAppendix. Surgical Procedures Considered and Corresponding International Classification of Diseases, Ninth Revision (ICD-9) Procedure Codes

Surgical Procedure	ICD-9 Code
Laparoscopic right hemicolectomy	17.33
Laparoscopic sigmoidectomy	17.36
Laparoscopic left hemicolectomy	17.35
Laparoscopic cecectomy	17.32
Other laparoscopic partial excision of large intestine	17.39
Laparoscopic resection of transverse colon	17.34
Laparoscopic multiple segmental resection of large intestine	17.31
Laparoscopic total intra-abdominal colectomy	45.81
Open and other right hemicolectomy	45.73
Open and other sigmoidectomy	45.76
Open and other left hemicolectomy	45.75
Open and other cecectomy	45.72
Open and other resection of transverse colon	45.74
Open and other multiple segmental resection of large intestine	45.71
Open total intra-abdominal colectomy	45.82

© 2019 Caroff DA et al. *JAMA Network Open.*
